# Plant-Derived Antioxidants: Significance in Skin Health and the Ageing Process

**DOI:** 10.3390/ijms23020585

**Published:** 2022-01-06

**Authors:** Monika Michalak

**Affiliations:** Department of Dermatology, Cosmetology and Aesthetic Surgery, Collegium Medicum, Jan Kochanowski University, IX Wieków Kielc 19, 35-317 Kielce, Poland; monika.michalak@ujk.edu.pl; Tel.: +48-41-349-6970

**Keywords:** antioxidants, skin, natural compounds, phytochemicals, polyphenols, tocopherols, carotenoids, ascorbic acid, macromolecules, essential oils

## Abstract

Natural substances have traditionally been used in skin care for centuries. There is now an ongoing search for new natural bioactives that not only promote skin health but also protect the skin against various harmful factors, including ultraviolet radiation and free radicals. Free radicals, by disrupting defence and restoration mechanisms, significantly contribute to skin damage and accelerate ageing. Natural compounds present in plants exhibit antioxidant properties and the ability to scavenge free radicals. The increased interest in plant chemistry is linked to the growing interest in plant materials as natural antioxidants. This review focuses on aromatic and medicinal plants as a source of antioxidant substances, such as polyphenols, tocopherols, carotenoids, ascorbic acid, and macromolecules (including polysaccharides and peptides) as well as components of essential oils, and their role in skin health and the ageing process.

## 1. Introduction

The world of plants is one of the main sources for materials used in the food, pharmaceutical and cosmetics industries. Many supplements, nutricosmetics and cosmetics are based on botanical ingredients, many of which have a long history of use in traditional or folk medicine [1,2]. Products of plant origin, including herbal teas, are consumed in many cultures for medicinal purposes, as well as for their taste attributes. Plant materials, including extracts, can also be applied topically for skin care purposes, as well as for treatment of many skin diseases [3]. In addition to the aromatic effects of plants, emphasis is also placed on their antioxidant properties and ability to modulate certain types of skin damage resulting from harmful environmental factors, including ultraviolet radiation (UVR) and free radicals [4].

Reactive oxygen species (ROS) comprise non-radical and free radical species. They may be formed by the incomplete reduction of oxygen molecules. The products of one-electron, two-electron, and three-electron reduction of oxygen are superoxide anion radical (O_2_^•−^), hydrogen peroxide (H_2_O_2_), and hydroxyl radical (^•^OH), respectively. ROS also include species such as singlet oxygen (^1^O_2_), peroxyl radicals (ROO^•^), alkoxyl radicals (RO^•^), and organic hydroperoxides (ROOH) [5,6,7,8]. ROS are formed during numerous biochemical processes taking place under physiological and pathological conditions, including aerobic metabolism, antimicrobial mechanisms, and inflammatory responses. They are also generated by the effects of physical factors such as alpha, beta, gamma, ultraviolet, visible, and X-radiation, ultrasound, or temperature, as well as by chemical compounds, including xenobiotics, pesticides, ozone, and cigarette smoke [6,8,9,10,11,12].

Free-radical reactions take place in nearly all cells of the human body. They mediate important cellular processes such as growth, proliferation, differentiation, and apoptosis [6,13]. Free radicals produced in excess exert direct destructive effects on cell components. A high level of free radicals in the absence of effective antioxidant mechanisms may cause extensive damage to cellular structures [6,12,13]. During oxidative stress (OxS), the balance between the generation of free radicals and their neutralization by the body’s defence mechanisms is disturbed [14]. OxS is an important factor in the pathogenesis of many diseases, such as atherosclerosis, diabetes, cataracts, bronchial asthma, Alzheimer’s disease, cancer, and rheumatoid arthritis [5,7,13]. The works of many authors also indicate that oxidative damage has a significant role in ageing processes [10,13,14].

The aim of the present review is to describe the role of free radicals and OxS in the physiology and ageing of the skin, and to discuss the role of selected bioactive compounds of plant origin in skin health and the ageing process.

## 2. OxS and Skin Ageing

Free radicals enter into chemical reactions with cell components with ease. Their action results in lipid oxidation, protein conversion, and damage to nucleic acid structures [6,14]. Lipids and proteins are the basic components of biological membranes; therefore, damage caused to them by free radicals can lead to changes in both the membranes surrounding cells and in intracellular membranes, which are an integral part of cell organelles such as the cell nucleus, mitochondria, endoplasmic reticulum, Golgi apparatus, lysosomes and peroxisomes [7,14]. The harmful action of free radicals in biological membranes leads to lipid peroxidation (LPO). LPO end products (aldehydes, ketones, and hydroxy peroxides) affect both membranes and cell components. OxS results in the disintegration of membranes and increases their permeability. Moreover, aldehydes formed during LPO have been shown to exert mutagenic and carcinogenic effects. One of the end products of LPO, produced in the highest amounts, is malondialdehyde (MDA), which is recognized as a measure of this process in the body. Lipid peroxidation products, including MDA and 4-HNE (4-hydroxynonenal), are believed to play a role in the initiation of protein oxidation. They can also damage nucleic acid molecules [7,12,14]. The hydroxyl radical is mainly responsible for DNA damage. Its reactions with nucleic acids can lead to damage to purine and pyrimidine bases and sugar residues or to breakage of phosphodiester bonds. Free radicals, by significantly contributing to DNA damage, lead to mutations and play an important role in carcinogenesis [12,14].

Skin cells are constantly exposed to the harmful effects of free radicals that are generated by both endogenous and exogenous factors [15]. Although the skin has natural defence mechanisms against free radicals, it is susceptible to their effects when they are produced in excessive amounts [11,16]. ROS affect the epidermis and dermis. Free radicals may damage the skin by destroying lipid components of sebum and ceramides of the intercellular cement of the stratum corneum or by oxidation of polyunsaturated fatty acids of cell membrane phospholipids [17,18]. Free radicals, including ^1^O_2_, directly damage the DNA and lipids of epidermal keratinocytes [19]. High ROS levels induce a complex cellular response in keratinocytes, with activation of the NF-κB (nuclear factor kappa-light-chain-enhancer of activated B cells) pathway. NF-κB is a kappa light-chain synthesis promoter in B cells associated with cellular longevity (regulating the expression of telomerase genes, inflammation, angiogenic and anti-apoptotic factors, and cellular proliferation) and is involved in the development of skin diseases (psoriasis vulgaris, allergic dermatitis, and skin cancer) [19,20]. In human keratinocytes, oxidative stress caused by ROS can also lead to the activation of mitogen-activated protein kinase (MAPK) pathways. Increased ROS production in a cell activates extracellular signal-regulated kinases (ERKs), c-Jun N-terminal kinases (JNKs), or p38 MAPK. Activated MAPKs phosphorylate various substrate proteins (e.g., transcription factors), resulting in regulation of various cellular activities (e.g., proliferation, differentiation, inflammatory responses, and apoptosis) [20]. Moreover, ROS contribute to OxS-induced degradation of melanocytes and compromise the function of cellular proteins, such as tyrosine-related protein 1 (TRP1), involved in melanogenesis [21]. ROS are also capable of inducing the expression of proteinases responsible for remodeling the extracellular matrix (ECM), such as serine proteases and matrix metalloproteinases (MMPs), mainly collagenase 1 (MMP-1). This enzyme is responsible for the degradation of collagen, which is the main building component of the skin. Moreover, oxidized lipids, such as linoleic acid hydroperoxide, enhance the expression of MMP-1 and MMP-3 [17,22,23]. Free radicals also damage elastin fibres and cause depolymerization of hyaluronic acid [17].

**Figure 1:**
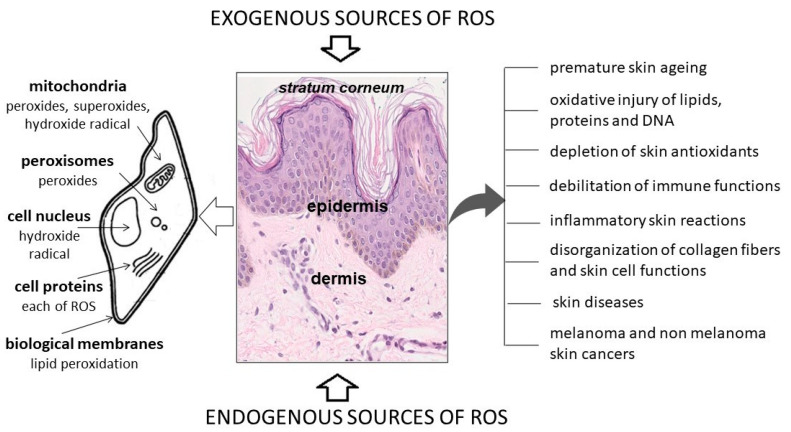
Potential cellular components attacked by reactive oxygen species (ROS) and the effect of oxidative stress on the skin (own work based on [6,7,17]; photo: Department of Clinical and Experimental Pathology, Collegium Medicum, Jan Kochanowski University).

Free radical reactions lead to skin lesions, which are characterized by a disruption of defence and restoration mechanisms in the skin [17,24]. Free radicals adversely affect the condition and functioning of the skin, and OxS is one of the major mechanisms of skin ageing (Figure 1) [22].

Skin ageing is a natural, complex process influenced by two mechanisms–intrinsic ageing (genetic, chronological) and extrinsic ageing (photoageing) [9,17,25]. Both intrinsic and extrinsic ageing are associated with changes in the physical, morphological, and physiological properties of the epidermis and dermis [8,9,11]. The two processes overlap and are strongly associated with an increase in free radicals and the phenomenon of OxS in the skin [9]. One of the main factors accelerating intrinsic skin ageing is oxidative damage to cellular structures. Skin ageing is also significantly accelerated by UVR [8,17,24,25]. Both UVA and UVB radiation are important sources of ROS formation in the skin. Photo-oxidative stress caused by ROS produced in the skin under the influence of solar radiation is believed to be the main pathological mechanism causing damage to ECM proteins (responsible for the formation of wrinkles) as well as photomutagenesis of skin cells (responsible for carcinogenesis). Chronic photo-oxidative stress causes symptoms of skin photoageing, including a reduction in the number of dermal fibroblasts, the formation of collagen cross-links, protease-induced collagen breakdown, and chronic inflammation. OxS may also increase the level of elastin mRNA in dermal fibroblasts, contributing to the elastotic changes found in skin exposed to UVR [11,17,25].

OxS results in accelerated wrinkle formation, loss of elasticity, dryness, uneven pigmentation and discoloration, telangiectasia, susceptibility to irritation, and slower wound healing [17].

OxS may also play an important role in numerous dermatological diseases, such as psoriasis, atopic dermatitis, allergic contact dermatitis, acne vulgaris, vitiligo, lichen planus, alopecia areata, and melanoma [22].

## 3. Antioxidants and the Skin

To counteract changes resulting from OxS, the body has developed many mechanisms to protect against the generation of free radicals and to convert them into inactive derivatives. These mechanisms include compounds of both exogenous and endogenous origin that form a complex antioxidant system. An antioxidant is a substance whose presence in low concentrations relative to the substrate susceptible to oxidation significantly lowers or prevents the harmful effects of free radicals on human tissues. Antioxidants are a chemically heterogeneous group of compounds that can be classified according to their structure, solubility (in water or fat), and the kinetics of the reactions in which they are involved. Fat-soluble antioxidants include α-tocopherol, β-carotene, lipoic acid, and ubiquinone (coenzyme Q10), while water-soluble antioxidants include glutathione (GSH) and ascorbic acid. We can distinguish enzymatic antioxidants, such as superoxide dismutase (SOD) (manganese SOD (MnSOD) in the mitochondria, copper and zinc SOD (Cu/Zn SOD) in the cytoplasm, and extracellular SOD), catalase (CAT), and GSH-dependent enzymes, i.e., glutathione peroxidase (GPx1–GPx8), glutathione transferase (GST) and glutathione reductase (GR), as well as non-enzymatic antioxidants: GSH, uric acid, melatonin, metal chelators (transferrin and lactoferrin), lipoic acid, ubiquinone, transition metal ions (zinc, copper, and selenium), vitamin E (vit E), vitamin C (vit C), β-carotene, and polyphenolic compounds (Table 1) [6,12,13,17].

Based on their mechanism of action, antioxidants can be classified as those that act on the levels of prevention, interception, and repair. Antioxidant enzymes (SOD, CAT and GPx) are preventive antioxidants, preventing the formation of ROS. Enzymatic proteins such as ceruloplasmin and haem proteins also play an important role in the fight against ROS. Interception of free radicals takes place mainly by radical scavenging. Here the most important role is played by low-molecular-weight antioxidants, such as vit C, vit E, GSH, α-lipoic acid, melatonin, carotenoids, and flavonoids. The third line of defence is repair of damage caused by ROS. It consists of antioxidant enzymes with oxidoreductase activity, which can reduce LPO products (paraoxonase), or disulphide bridges formed as a result of DNA peroxidation (thioredoxin (TRX) or GPx) [6,12,13].

The antioxidant defence mechanism plays an important role in protecting the skin against oxidative damage [15,17,22,26]. The skin is equipped with mechanisms aimed at combating free radicals and interrupting radical reactions [15,17]. The concentration of antioxidants is higher in the epidermis than in the dermis [9]. The stratum corneum contains non-enzymatic hydrophilic and lipophilic antioxidants, such as GSH, ascorbic acid, uric acid, α-tocopherol, squalene, and ubiquinone [15,17]. The individual layers of the epidermis contain lipophilic antioxidants, especially α-tocopherol, as well as enzymes such as SOD, CAT, and GPx [17,22,26]. The dermis contains water-soluble non-enzymatic antioxidants, such as vit C, uric acid, and GSH, as well as antioxidant enzymes [17].

Defence against free radicals often involves the interaction of hydrophobic and hydrophilic antioxidants, or of enzymes with non-enzymatic antioxidants. However, these processes may be insufficient when exposure to oxidizing agents is excessive [9,15,26]. In vivo studies have shown that during intrinsic ageing and photoageing processes certain changes take place in major antioxidant enzymes and antioxidant molecules in the human epidermis and dermis. CAT activity has been found to be significantly increased in the epidermis of photoaged and naturally aged skin, while GR was significantly higher in naturally aged epidermis. The concentration of α-tocopherol was significantly lower in the epidermis of photoaged and aged skin, while ascorbic acid levels were lower in both the epidermis and dermis of photoaged and naturally aged skin [27].

Among antioxidant substances, those derived from plants play an important role in preventing and repairing skin damage caused by free radicals. Plant extracts not only have the ability to scavenge free radicals, but also support the defence and regenerative mechanisms of the skin [8,9,17,18,22,24].

## 4. The Antioxidant Activity of Plants

Plants are a rich source of biologically active substances that have a significant effect on human skin [28]. The biological activity of plants and the content of active ingredients in them are mainly influenced by the environment in which they develop, the time of harvesting, and the conditions in which the herbal material is dried and stored [29,30]. Plants can exhibit a variety of properties, both medicinal in the case of certain skin diseases and promoting skin health, including through antioxidant effects [24,28]. The free-radical scavenging ability and antioxidant properties of plants are associated with the presence of components such as polyphenols, tocopherols, carotenoids, ascorbic acid, and macromolecules (including polysaccharides and peptides), as well as components of essential oils [16,24,31].

To assess the antioxidant properties of plants, plant extracts and their components there are used such chemical-based methods as: (1) radical scavenging assays (DPPH (2,2’-diphenyl-1-picrylhydrazyl), ABTS (2,2’-azinobis-(3-ethylbenzothiazoline-6-sulfonic acid), and hydroxyl assays), lipid peroxidation assays (β-carotene-linoleate model systems, thiobarbituric acid reactive substances (TBARS) assays), and reduction power assays (the ferric antioxidant power reduction (FRAP), cupric ion reducing antioxidant capacity (CUPRAC), phosphomolybdenum (PM) assays), as well as (2) cell- and enzyme-based assays such as cellular antioxidant activity (CAA) assay and inhibition of antioxidant enzymes [32,33,34].

### 4.1. Polyphenols

Polyphenols are a very large and important group of natural compounds commonly found in the plant world. Polyphenols are organic chemical compounds containing two or more hydroxyl groups bound to an aromatic ring. Depending on their chemical structure, they can be classified as flavonoids, phenolic acids, tannins, or stilbenes (Table 2) [35,36]. Flavonoids are the best-known group of polyphenols. They can exist as free molecules, known as aglycones, or more commonly in a form bound to sugars, as glycosides [35]. Important sources of flavonoids include onions, leek, chicory, rocket lettuce, fresh capers, radish, sorrel, blackcurrants, goji berries (flavonols); parsley, celery, kohlrabi, oregano, artichokes, capsicum pepper (flavones); green tea, apples, cherry, peach, apricot, pecan nuts, beans (flavanols); citrus fruits (flavanones); soybeans (isoflavones). Anthocyanins constitute an important group of flavonoid compounds with the ability to scavenge free radicals. The most common anthocyanin pigments include red pelargonidin (geranium, dahlia), purplish red peonidin (elderberry, peony) and cyanidin (cornflower, chokeberry, cranberry, sour cherry), and purple malvidin (mallow, grapes), petunidin (petunia) and delphinidin (grapes, elderberry, cranberry). Phenolic acids are the second group of polyphenolic compounds with antioxidant properties. Hydroxycinnamic acids are more common than hydroxybenzoic acids, which can be found in fruits such as blackberry, raspberry, and blackcurrant. Hydroxycinnamic acids are found in all parts of the fruit, although the highest concentrations are found in the outer parts of ripe fruits such as blueberry, kiwi, cherry or plum [37,38,39].

The diversity of polyphenol structures is linked to their multi-faceted biological activity. Polyphenolic compounds, in addition to their anti-inflammatory, moisturizing, smoothing, soothing, anti-ageing, UV-protective, antibacterial, and capillary stabilizing properties, exert a strong antioxidant effect [16,23,35,43,44]. The antioxidant activity of phenolic compounds, resulting from various mechanisms of action, consists of (1) eliminating reactive oxygen species through direct reaction with free radicals, scavenging of free radicals, and enhancing dismutation of free radicals to compounds with much lower reactivity; (2) inhibiting or potentiating the action of numerous enzymes, e.g., oxidases, and increasing the expression of antioxidant proteins such as CAT and SOD; (3) chelating pro-oxidative metal ions (e.g., iron or copper); and 4) enhancing the effect of other antioxidants (e.g., restoring the original form of tocopherols from the radical form or prolonging the action of ascorbic acid) [42,45].

Polyphenols with strong antioxidant potential include myricetin, quercetin, catechin, kaempferol, resveratrol and ferulic acid [35].

Results from previous studies indicate that the anti-radical and antioxidant activity of plant extracts is linked to the content of polyphenolic compounds in raw plant material (Table 3).

Polyphenolic raw materials with antioxidant properties include fruits, vegetables, fruit juices, herbs, and spices, as well as oil seeds. Phenolic compounds can also be supplied to the body in the form of plant extracts as drugs, dietary supplements, nutricosmetics or cosmetics [16,23,43]. In the case of oral supplements, the bioavailability of polyphenols, which largely depends on their chemical structure, is of great importance. The biological activity of cosmetics enriched with plant polyphenols depends on their ability to penetrate the skin barrier. In the case of topical preparations, the release of the active substance from the cosmetic form and its ability to reach the skin and finally penetrate through the stratum corneum deep into the epidermis or dermis are of particular importance. The beneficial effects of polyphenols on the skin have resulted in the wide application of plant extracts containing this group of compounds in numerous skin care products [23,46].

### 4.2. Carotenoids

Carotenoids are a class of fat-soluble natural pigments, responsible for the colour of many plants, including fruits, vegetables, and flowers. Carotenoids include two main classes, namely, carotenes (consisting of carbon and hydrogen), including α-, β-, and γ-carotene and lycopene, and their oxygenated derivatives, xanthophylls (consisting of carbon, hydrogen, and oxygen), including lutein, zeaxanthin, astaxanthin, and β-cryptoxanthin [47,48].

**Figure 2:**
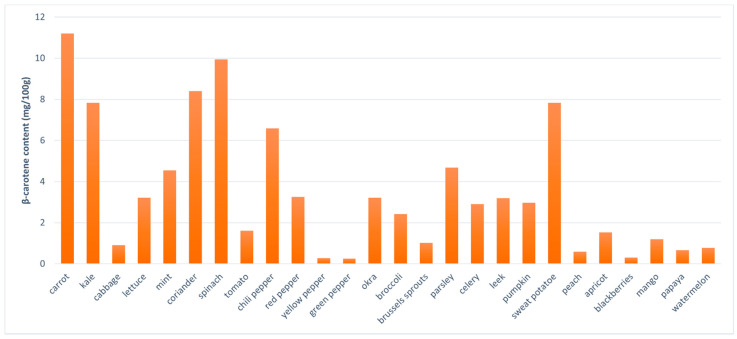
Selected plant sources of β-carotene [51,52,53].

Sources of natural carotenoids include plants such as carrots (e.g., α- and β-carotene and lycopene), chili pepper (capsorubin and capsanthin), tomato (β-carotene and lycopene), broccoli (β-carotene and lutein), spinach (neoxanthin and lutein), pumpkin (β-carotene, β-cryptoxanthin, lutein, and zeaxanthin), watermelon (lycopene, α-, β- and γ-carotene), apricot (β-carotene and lycopene), papaya (β-carotene, β-cryptoxanthin, and lycopene), sea buckthorn (α-, β-, γ-carotene, lycopene, cryptoxanthin, lutein, and zeaxanthin) and dog rose (β-carotene, lutein, and lycopene). β-Carotene is the most widely distributed carotenoid in foods, found mainly in yellow-orange and dark green fruits and vegetables (Figure 2) [38,48,49,50].

Carotenoids, as natural antioxidants that protect cellular lipids, proteins, and DNA from attack by free radicals, play a key role in maintaining human health, including skin health. The antioxidant power of carotenoids is linked to the high number of conjugated double bonds in their structure and their lipophilicity. Carotenoids such as lycopene, α-, β-, and γ-carotene, β-cryptoxanthin, lutein, and zeaxanthin are found in the epidermis, dermis, and subcutaneous fat [54,55]. Research results suggest that β-carotene and lycopene are present in greater amounts than zeaxanthin and lutein in human skin [56]. Carotenoids such as β-carotene, lycopene, and lutein are of prime importance in reducing the risk of skin cancer development and skin ageing (Table 4) [57].

The content of carotenoids in human skin varies, with the highest levels found in parts of the body with a high concentration of sweat and with sebaceous glands. The total content of carotenoids in the skin is influenced by many factors, such as season, exposure to UV radiation, and intake of fruit, vegetables, and carotenoid-rich supplements, as well as air pollution, alcohol consumption, cigarette smoking, stress, and the use of cosmetics containing provitamin A [55]. The external application of preparations containing carotenoids in combination with oral supplementation has been shown to increase the concentration of these compounds in the skin [43,63]. Research results showed that a combined oral and topical administration of lutein and zeaxanthin improves skin physiology parameters, including surface lipids, hydration, photoprotective activity, skin elasticity and skin LPO (MDA) in human subjects. The oral administration of lutein was shown to provide a higher level of antioxidant protection than topical application of this antioxidant. Research has shown a significant reduction in UV-induced erythema and better protective activity in the skin, measured as changes in lipid peroxidation, following oral application of lutein [64]. Several human studies also indicate that supplementation with β-carotene (alone, in combination with vit E, or as a carotenoid mixture consisting of β-carotene, lutein and lycopene) has a photoprotective role and alleviates UV-induced erythema [65,66]. Other studies have shown that the oral administration of β-carotene, lycopene, α-tocopherol, and selenium reduces UV-induced erythema, LPO, and sunburn cell formation [67]. It should be emphasized that these effects may not be attributable to a direct effect of the phytochemicals on skin cells, which may not have been selectively targeted by the phytochemicals, but due to their effects in maintaining the overall health of the body. However, β-carotene supplements may be used as oral sun protectants for the skin [54]. Due to the ability of β-carotene to scavenge radicals such as singlet oxygen or hydroxyl radical, it plays an important role in the treatment of photodermatoses caused by UVR [38]. According to the research findings reported thus far, carotenoid supplementation has beneficial results, especially in protection against UVR and ROS [55,57].

### 4.3. Tocopherols and Tocotrienols

Vit E is present in eight isoforms: α-, β-, γ-, and δ- tocopherol, and α-, β-, γ-, and δ- tocotrienol. These molecules consist of a hydrophobic prenyl group that penetrates the cell membrane and a polar chromanol ring on the surface of the cell membrane. Tocopherols and tocotrienols differ only in their prenyl residues [68,69].

The most active form of vit E found in humans is α-tocopherol [69]. It is believed that α-tocopherol primarily inhibits the production of new free radicals, while γ-tocopherol traps and neutralizes existing free radicals [70]. Vit E is involved in various physiological and biochemical functions of the body. It is highly important as a membrane stabilizer and a lipid-soluble antioxidant. Tocopherols play key roles in protecting cellular membranes against LPO by free radicals. Vit E also promotes membrane repair by preventing the formation of oxidized phospholipids. The mechanism of the antioxidant activity of tocopherols is linked in part to the presence of a hydroxyl group in the chromanol ring, which donates a hydrogen atom to reduce free radicals [11,12,13,69,70]. The antioxidant reaction results in the oxidized form of α-tocopherol, which can be reduced by ascorbic acid [71].

The antioxidant properties of vit E play an important role in combatting various diseases, such as atherosclerosis, cancer, cataracts, Alzheimer’s disease, and cardiovascular diseases [70]. The role of vit E in the protection against photoageing and skin cancer is also significant [68]. The stratum corneum, which is the external protective structure defending against the harmful effects of OxS caused by solar radiation, contains an exceptionally high level of vit E [72]. α-Tocopherol is the major antioxidant in the human epidermis, and its depletion is an early and sensitive marker of environmental oxidative damage [73]. A study using a topical vit E analogue, Trolox (6-hydroxy-2,5,7,8-tetramethylchroman-2-carboxylic acid), showed inhibition of UVB-induced intracellular peroxide generation in human keratinocytes [74]. Due to its lipophilic properties and the affinity of tocopherols for the lipids of intercellular cement, vit E is well absorbed when applied topically. Hence, it is an important skin care ingredient with antioxidant properties [68]. An important feature of vit E that makes it especially valuable in skin protection is its ability to become incorporated in the structures of the cell membrane and lipids of intercellular cement. Owing to this location, vit E is present in areas of the skin that are particularly vulnerable to damage. Topical application of this group of antioxidants has been shown to reduce acute and chronic photodamage. Vit E reduces the harmful collagen-destroying enzyme collagenase, inhibits tyrosinase, and also reduces skin roughness and the depth of wrinkles. Moreover, it accelerates healing and therefore is used in the treatment of burns, surgical scars, and wounds [4,68].

**Figure 3:**
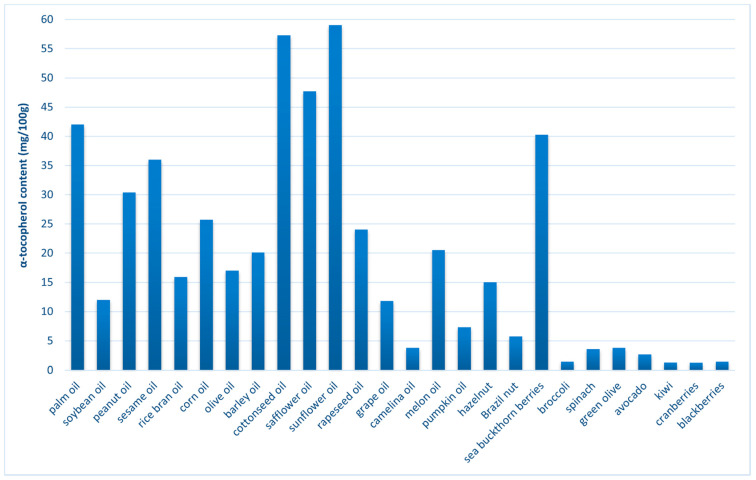
Selected plant sources of α-tocopherol [70,75,76,77,78].

This vitamin can be found in vegetable oils (especially wheat germ), nuts (especially almonds), seeds (such as sunflower seeds), greens (such as spinach) and whole grains (Figure 3) [68,70].

### 4.4. Ascorbic Acid

Vit C is an important and highly efficient water-soluble antioxidant. Because ascorbic acid can donate two electrons, it is a cofactor in numerous enzymatic reactions in the body [79]. Owing to its antioxidant capacity, vit C protects the cells of the body, including skin cells, against OxS [11,79]. Ascorbic acid is oxidized and transforms into the ascorbate anion. The ascorbate anion can continue the electron donation, leading to conversion to ascorbate free radical, which in turn is converted to dehydroascorbic acid [79]. Due to the successive electron donations, the resulting ascorbate free radical has greater stability than other free radicals and can serve as an antioxidant scavenging other radicals. Although the direct antioxidant protection provided by vit C is limited to aqueous compartments, vit C significantly prevents LPO by regenerating fat-soluble vit E [79,80]. Furthermore, vit C interacts with carotenoids and antioxidant enzymes. Ascorbic acid increases the concentration of intracellular GSH, which protects protein thiol groups against oxidation [71,81].

Vit C is essential for maintaining the appropriate structure and function of the skin. Ascorbic acid is found in all layers of the skin, with a higher concentration in the epidermis (3.8 µmol/g) than in the dermis (0.7 µmol/g) [72]. It stimulates the synthesis of ceramides–lipid compounds found in the stratum corneum that are responsible for maintaining proper skin hydration [82]. Ascorbic acid, through its participation in the formation of collagen cross-links during the hydroxylation of proline and lysine, is involved in the synthesis of collagen I and III. It also inhibits melanogenesis and the enzyme tyrosinase [11]. Topical application of vit C significantly prevents oxidative damage caused by UVR [83].

**Figure 4:**
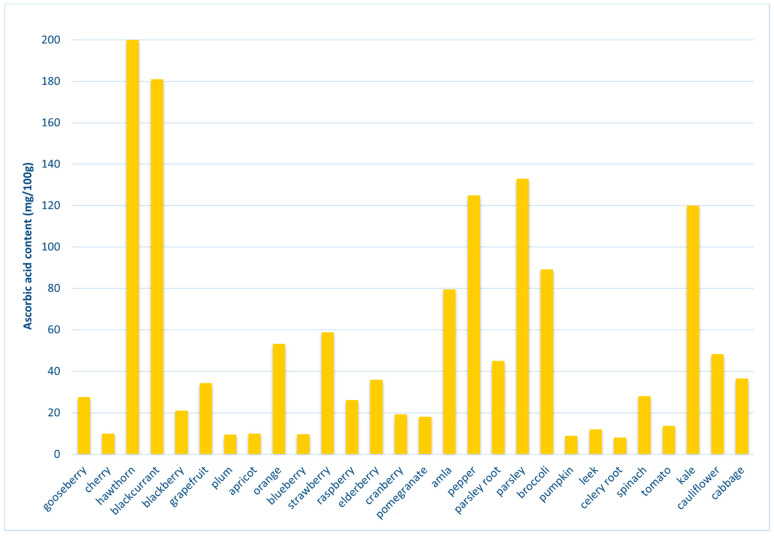
Selected plant sources of ascorbic acid [79,86,87,88,89].

Fresh vegetables and fruits are excellent sources of vit C (Figure 4), especially dog rose (250–800 mg/100 g), sea buckthorn (695 mg/100 g), goji berries (716.91 mg/100 g), blackcurrant (150–300 mg/100 g) and red pepper (125–200 mg/100 g) [67,79,84,85].

### 4.5. Macromolecules

Macromolecules, such as proteins, peptides, and polysaccharides, are claimed to be health-promoting agents which can be used in medicine and in the cosmetics and food industries [31,90]. These exogenous bioactive compounds serve as important organic components of the body and can regulate the redox state. Biomacromolecules can effectively remove ROS to slow ageing, protect skin cells, inhibit melanin production, and prevent lipid oxidation, especially in polyunsaturated fat-based products [31,91]. Several exogenous bioactive macromolecules have been shown to occur widely in plants [31].

There is growing interest in peptides from plant resources. They can be obtained from herbs, fruits, seeds or leaves [90,91]. Bioactive peptides (BPs) are defined as specific protein fractions (sequence of 2–20 amino acids) with positive physiological functions [31,91]. The consumption of food-derived peptides can supplement endogenous enzymatic and non-enzymatic antioxidant systems and effectively reduce oxidative stress [90]. While the exact mechanisms of action of antioxidant peptides are not fully understood, some studies have demonstrated that BPs exhibit antioxidant properties through a variety of mechanisms, including scavenging of free radicals, electron or hydrogen transfer reduction, transition metal chelating activity, and ferric reducing power, as well as prevention of LPO [31,91]. Moreover, these molecules can also exhibit antioxidant activity by inducting the gene expression of proteins that protect cellular components from oxidative stress-induced damage. The antioxidant activities of peptides depend on their structural properties (e.g., amino acid composition (amount of histidine, cysteine, proline, methionine, or aromatic amino acids), molecular size, and hydrophobicity) but also on the nature of the ROS [90]. For instance, histidine residues of peptides can chelate metal ions, quench active oxygen, and scavenge ^•^OH; phenylalanine can scavenge ^•^OH radicals to form more stable *para*-, *meta*-, or *ortho*-substituted hydroxylated derivatives; the electron-dense aromatic rings of phenylalanine, tyrosine, and tryptophan residues of peptides can contribute to the chelating of pro-oxidant metal ions, while hydrophobic amino acids such as valine, tyrosine, methionine or phenylalanine at the C-and N-termini may promote the antioxidant activity of peptides [91].

The available literature provides data on plant sources of antioxidant bioactive peptides, including lentil, chickpea, pea, beans, soy, oat, wheat, maize, rice, barley, millet, sorghum, peanut, walnut, pine nut, rape, sesame, hemp, flax, amaranth, chia, pumpkin, watermelon, perilla, moringa, sunflower, cassia, alfalfa, mulberry, sweet potato, African legume crops, and others [31,90,91]. Delgado et al. demonstrated that amaranth (*Amaranthus mantegazzianus*) seeds contain naturally-occurring peptides and polypeptides, which can scavenge free radicals and inhibit linoleic acid oxidation [92]. An in vitro study by Phongthai et al. demonstrated the DPPH and ABTS radical-scavenging activity and ferric reducing antioxidant power in peptides found in rice bran [93]. Chai et al. reported strong ABTS scavenging and iron chelating activities for semen cassiae (*Cassia obtusifolia*) protein hydrolysate [94]. Evangelho et al. demonstrated the antioxidant properties of black bean (*Phaseolus vulgaris*) protein hydrolysates, measured in vitro, by DPPH and ABTS scavenging activity [95]. Cheng et al. found that cooperativity between BPs from potato (rich in leucine, tyrosine, methionine, and phenylalanine) and surfactants (Tween 20) contributes to the oxidative stability of oil-in-water emulsions [96]. These examples demonstrate that peptides are promising antioxidants in the development of functional foods and nutraceuticals.

For the food and cosmetics industry, the use of polysaccharides is also interesting. Polysaccharides are among the important bioactive macromolecules widely distributed in plants. These high-molecular-weight polymers, composed of at least 10 monosaccharide molecules connected by glycosidic bonds, are essential for cell proliferation and the growth and development of organisms. Methods for the preparation of plant polysaccharides include hydro-alcohol precipitation, fermentation-alcohol precipitation, microwave extraction, ultrasonic extraction, enzymatic extraction, ultrasonic-assisted enzyme extraction, and vacuum extraction [97]. Polysaccharides are among the active ingredients of many medicines and exert numerous beneficial pharmacological effects, including antioxidant and anti-ageing properties [31,97]. They increase antioxidant enzyme activity, scavenge free radicals, and inhibit LPO [97].

The antioxidant activity of the polysaccharides of medicinal plants such as *Arctium lappa*, *Althaea officinalis*, *Plantago lanceolata*, *Rudbeckia fulgida*, *Mahonia aquifolium*, *Prunus persica, Angelica sinensis, Codonopsis pilosula, Lycium barbarum, Polygonum multiflorum,* *Astragalus membranaceus*, *Aloe vera, Portulaca oleracea, Amorphophallus konjac, Dioscorea opposita*, and Panax japonicus has also been evaluated [97,98]. Luo et al. showed that polysaccharide from *Polygonum multiflorum* isolated by water extraction and ethanol precipitation has powerful scavenging abilities, especially against ABTS, DPPH and ^•^OH, and suggest that this compound should be explored as a novel potential antioxidant [99]. A study by Pu et al., exploring carbon tetrachloride (CCl4)-induced liver damage in mice, found that *Astragalus membranaceus* polysaccharide significantly decreased MDA activity and enhanced SOD activity in these mice [100]. Research results by Wang et al. indicated that polysaccharide from *Gynostemma pentaphyllum* eliminated superoxide anions and DPPH and ABTS free radicals and also enhanced SOD, CAT, and GPx activity, while decreasing MDA activity [101]. An in vitro study by Ju et al. reported that Chinese yam polysaccharide isolated from the rhizomes of *Dioscorea opposita* exhibited notable scavenging activity against DPPH, ^•^OH and O_2_^•−^ radical [102]. Another important purpose of the addition of polysaccharides is to enhance the oxidative stability of emulsions containing fatty acids, β-carotene, anthocyanins and other bioactive components [103].

Owing to their wide distribution and high bioactivity, specificity and safety, antioxidant bioactive macromolecules are promising potential health-promoting agents for the prevention of various diseases.

### 4.6. Components of Essential Oils

Essential oils (EOs) are natural products derived from raw plant materials, including flowers (clove, turmeric, lavender, orange), leaves (rosemary, marjoram, oregano, basil, mint, parsley), roots (turmeric, ginger), seeds (coriander, cumin, fennel), fruits (peppers, star anise, tamarind), wood (sandalwood, rosewood), bark (cinnamon), and resin (myrrh) for their various biological properties and medicinal uses [32,104,105]. Many medicinal and aromatic plants belong to *Lamiaceae,* *Piperaceae,* *Apiaceae, Rutaceae,* *Pinaceae, Zingiberaceae, Asteraceae, Rosaceae, Myrtaceae, Lauraceae*, and *Verbenaceae* families [105,106]. EOs are volatile substances, mostly colorless or light yellow, of intense odor and oily consistency, soluble in liquid fats, alcohol, ether or chloroform. They are obtained by steam distillation, solvent extraction, Soxhlet extraction, microwave-assisted extraction, solid-phase microextraction, dry distillation of wood, squeezing of fruit peels, or transfer of the essential oil from flower petals to fat (enfleurage). The chemical composition, as well as the biological activity and fragrance, of oils depend on numerous factors, including physiological (plant organ, ontogenesis), environmental (soil composition, weather conditions), and genetic factors, as well as plant parameters (e.g., species, cultivated or wild plants) and other parameters such as storage conditions of EOs or their aging resulting from exposure to oxygen and UV light [106].

EOs are a mixture of many single organic substances, including terpene hydrocarbons and their oxygen derivatives, alcohols (geraniol, α-bisabolol), aldehydes (citronellal, sinensal), ketones (menthone, *p*-vetivone), esters (γ-tepinyl acetate, cedryl acetate). In general, one to three substances predominate in a particular oil, accompanied by a variety of complementary substances in trace amounts [32,104,105,106,107]. The dominant ingredients found in medicinal and aromatic plants include limonene (mint, hyssop, lavender, marjoram, oregano, thyme, vervain), linalool (mint, balm, basil, caraway, hyssop, lavender, marjoram, oregano, sage, thyme, vervain), citronellal (balm, sage, thyme), α-terpinene (mint, balm, hyssop, marjoram, oregano, thyme), pinene (dill, balm, basil, marjoram, oregano, sage), carvone (dill, coriander), thymol (marjoram, oregano, thyme, balm), myrcene (dill, parsley, mint, balm, basil, caraway, fennel, hyssop, lavender, marjoram, oregano, sage), *p*-cymene (caraway, fennel, lavender, marjoram, oregano, sage, thyme) [105].

Both medicinal and aromatic plants are valued for their antiseptic, antimicrobial, antifungial, anti-inflammatory, immunostimulating, neuroprotective, as well as antioxidant properties [32,105,107]. Terpenoids, i.e., small, fat-soluble organic molecules, contained in EOs, can penetrate the nasal mucosa if inhaled, and they can also penetrate the skin after topical application, as well as enter the blood and cross the blood-brain barrier [108]. Organic compounds contained in the EOs play an important role in scavenging free radicals and reducing OxS due to the conjugated carbon double bonds and hydroxyl groups present in their structure that can donate hydrogen. The most important components of EOs with antioxidant properties include thymol, carvacrol, geraniol, *p*-cymene, menthol, linalool, citronellal, isomenthone, menthone, α-terpinene, β-terpinene, and α-terpinolene [32,107].

In addition to the widespread use of EOs as flavoring substances, they are applicable as antioxidants in food, pharmaceutical and cosmetic industries [106,109]. Being a rich source of biologically active compounds, EOs are of growing interest as additives in the food products [104]. Research indicates the possibility of using EOs (oregano and thyme) as an alternative form of food packaging to improve food safety and quality [110]. The research also demonstrates that EOs could be used as a potential source of safe and natural antioxidant agents in the cosmetics as well as pharmaceutical products [106,111]. EOs are bioactive substances capable of scavenging free radicals, which damage proteins, carbohydrates, polyunsaturated fatty acids, and DNA, can limit the development of such degenerative processes and diseases as aging, immunodeficiencies, neurologic disorders, inflammation, and certain cancers [32,108,112,113,114]. Their neuroprotective and anti-ageing potential was studied with regard to EOs obtained from such plants as *Nigella sativa, Acorus gramineus, Lavandula angustifolia, Eucalyptus globulus, Mentha piperita, Rosmarinus officinalis, Jasminum sambac, Piper nigrum* [112]. Due to their antioxidant properties (mainly by reduction in ROS and the reduction in NF-κB reducing the expression of proinflammatory cytokines), a study was conducted that demonstrated the significant effect of oils in the treatment of acute and chronic inflammation found in plants such as *Oenanthe crocata, Callitris intratropica, Citrus reticulata, *Hibiscus* sabdariffa, Chamaecyparis obtusa, Citrus aurantium, Piper nigrum, Choisya *ternata**, *Nigella sativa* [114]. Moreover, *Eugenia caryophyllata* essential oil, with its major active component, i.e., eugenol, has anti-inflammatory and tissue remodelling properties (it inhibits the increased production of proinflammatory biomarkers such as vascular cell adhesion molecule 1 (VCAM-1), interferon γ-induced protein 10 (IP-10), interferon-inducible T-cell α chemoattractant (I-TAC), and monokine induced by interferon-gamma (MIG), as well as such protein molecules as collagen-I, collagen-III, macrophage colony-stimulating factor (M-CSF), and tissue inhibitor of metalloproteinase 2 (TIMP-2)) in human dermal fibroblasts [113]. It was also demonstrated that the treatment of healthy human keratinocytes (HaCaT) with EOs such as oregano, thyme, clove, arborvitae, cassia, lemongrass, melaleuca, eucalyptus, lavender, and clary sage increased the total antioxidant status (TAS) level [111]. Furthermore, *Origanum vulgare* essential oil is characterized by radical scavenging ability, anti-lipid peroxidation efficacy, as well as significant anti-skin-ageing properties, including inhibition of collagenase, elastase, and hyaluronidase [115].

Volatile composition and antioxidant properties were reported with regard to EOs derived from such plants as cinnamon, caraway, lemon, clove, tea tree, spearmint, basil, oregano, perilla, black pepper, patchouli, rosemary, sage, thyme, and ginger (Table 5).

### 4.7. Plants with Antioxidant Properties as Bioactives in Skin Care and Treatment

Plant extracts are currently among the most common ingredients incorporated into to skin care products. An advantage of this group of products is their mild and safe but effective action [3,28]. Plant extracts, as a rich source of biologically active substances, constitute an essential group of multifunctional skin care ingredients [16,24,28]. Their ability to improve the condition and appearance of the skin and their potential use in the treatment of various skin diseases, including phototoxicity, psoriasis, and atopic dermatitis, is well known [3,30,67]. Plant extracts exhibit moisturizing, nourishing, capillary-stabilizing, cleansing, anti-inflammatory, antimicrobial, emollient, melanin-inhibiting, antimutagenic, astringent, regenerating and UV-protective properties. The biological activity of plant extracts is also associated with antioxidant activity [30,67]. The topical application of antioxidants, including plant extracts, supports the endogenous defence mechanisms of the skin, helping to reduce UVR-mediated oxidative damage and prevent OxS-mediated diseases [124,125].

The literature includes some reports evaluating the impact of plant-derived antioxidants on skin. Lima et al. tested curcumin, a component of *Curcuma longa* rhizome, for its potential anti-ageing effects. Their in vitro study on normal human skin fibroblasts showed that curcumin induces cellular stress responses through the phosphatidylinositol 3-kinase/Akt pathway and redox signalling, supporting the theory that curcumin-triggered cellular antioxidant defenses can serve as an effective approach to anti-ageing therapy [126]. Katiyar et al. showed that a pre-treatment of normal human epidermal keratinocytes with epigallocatechin gallate (EGCG), an antioxidant derived from *Camellia sinensis*, inhibits UVB-induced H_2_O_2_ (oxidative stress) production and H_2_O_2_-mediated phosphorylation of MAPK signalling pathways (ERK1/2, JNK, and p38 were found to be significantly inhibited). This study demonstrates that EGCG could be useful in alleviation of oxidative stress-mediated and MAPK-caused skin disorders [127]. Another study confirmed that EGCG exerts photoprotective effects against solar UV radiation and enhances skin tolerance to UV-induced stress. The oral administration of EGCG to female HWY/Slc hairless rats for 8 weeks was shown to significantly increase the minimal UV-induced erythema dose (MED) and to protect against alterations in epidermal barrier function [128]. In another study, a *Vitis vinifera* proanthocyanidin extract exhibited 78–81% inhibition of superoxide anion and hydroxyl radical and was found to be a more potent scavenger of oxygen free radicals than vit C [129]. Foncesa et al. evaluated the in vivo protective effect of marigold extract against UVB-induced oxidative stress in the skin of hairless mice by determining reduced glutathione (GSH) levels. The results indicated that prevention of UVB irradiation-induced GSH depletion by oral treatment with 150 and 300 mg/kg of *Calendula officinalis* extract might be an important strategy in protection against UVB-induced skin damage [130]. Zaid et al. studied the effect of polyphenol-rich pomegranate fruit extract (10–40 μg/mL) on UVB-induced oxidative stress and photoageing in human immortalized HaCaT keratinocytes. The extract was shown to inhibit the UVB-mediated decrease in cell viability, decrease in intracellular glutathione content, and increase in lipid peroxidation, which indicates that *Punica granatum* extract may be a useful component in skin care products [131]. Widyarini et al. examined the potential of isoflavones from *Trifolium pratense* and some metabolically related compounds (equol, isoequol, and dehydroequol) to protect hairless mice against UV irradiation when applied topically after UV exposure. The results showed that equol markedly reduced UV-induced inflammation, suggesting that lotions containing equol, by protecting the immune system from photo-suppression, may play a role as sun-protective cosmetic ingredients in the future [132]. Wei et al. reported that genistein, the principal isoflavone present in soybeans, when applied daily to female mouse skin, is capable of inhibiting 7,12-dimethylbenz[a]anthracene (DMBA)-initiated and 12-O-tetradecanoyl phorbol-13-acetate (TPA)-promoted skin tumorigenesis. The authors suggest that genistein exerts anti-initiational and anti-promotional effects on skin carcinogenesis, probably through the inhibition of oxidative and inflammatory events in vivo [133]. Another in vivo study evaluated the impact of subacute and chronic UVB exposure on ROS production and oxidative damage to macromolecules such as lipids and DNA. The results showed that the topical application of genistein (10 µmol) significantly inhibited UVB-induced H_2_O_2_ production (by more than 50%) and also substantially inhibited the formation of MDA (by about 30% in mice exposed to UVB radiation for a week and more than 50% in mice exposed to UVB for 2 weeks) and 8-hydroxy-20-deoxyguanosine (8-OHdG) in mouse epidermis exposed to UVB. This experiment revealed that genistein exhibits potent antioxidant properties and explains the mechanisms of the anti-photocarcinogenic action of genistein [134]. Meinke et al. evaluated the free radical scavenging activity (in vitro in HaCaT keratinocytes irradiated with solar simulated radiation) of hyperforin, a major constituent of *Hypericum perforatum*, as well as the photoprotective effect of a cream containing 1.5% hyperforin-rich St. John’s wort extract. Hyperforin proved to be a much more effective free radical scavenger than Trolox or N-acetylcysteine, without showing phototoxicity. The cream significantly reduced radical formation following infrared irradiation and reduced UVB-induced erythema, which may explain the anti-inflammatory and UV-protective effects of hyperforin [135]. An in vitro study by Almeida et al. showed that *Juglans regia* leaf extract exerted scavenging effects on ROS (^•^OH, O_2_^•−^, ROO^•^, and H₂O₂) and RNS (NO and ONOO⁻) and can be used as a source of natural antioxidants [136].

Plant extracts from herbs, leaves, flowers, fruits, and seeds form an important group of botanical-based cosmetics known as cosmeceuticals. The components of these formulations, especially antioxidants that prevent skin ageing and that possess photoprotective properties, can also be used in oral supplements, referred to as nutraceuticals (Table 6) [1,3].

## 5. Conclusions

Plant products have been used for centuries for skin care purposes. Today, the use of natural ingredients in various innovative formulations for skin care, cleaning, and protection remains very popular. Both single active compounds and sets of compounds that are present in plants are used for therapeutic and cosmetic purposes, usually in the form of extracts obtained from various parts of the plant. Plant extracts are used because they can protect the skin against harmful exogenous or endogenous factors. The main benefits of natural ingredients include their antioxidant properties and their ability to prevent skin disorders resulting from OxS. The UV-protective effect of plant extracts is also important, as UV-induced photo-oxidative damage to cellular lipids, proteins and DNA is associated with premature skin ageing and the development of skin cancer. Plants have a great potential to support skin care, but more research trials and clinical evidence are needed, as the effectiveness of many such extracts has not yet been confirmed. Furthermore, many active molecules have yet to be discovered, and natural molecules derived from plant extracts are a particularly interesting subject for further research. Many new aromatic and medicinal plants that improve the quality of plant-based products may be identified in future.

## Figures and Tables

**Table 1:** Mechanisms of action of selected enzymatic and non-enzymatic antioxidants [12,13,17,19].

**Enzymatic Antioxidants**	
Superoxide dismutase (SOD)	- requires a metal as a cofactor (is a metalloenzyme) - catalyzes the dismutation of O_2_^•−^ into O_2_ and H_2_O_2_
Catalase (CAT)	- uses iron or manganese as a cofactor - catalyzes H_2_O_2_ into O_2_ and H_2_O
Glutathione peroxidase (GPx)	- is an important intracellular enzyme - its activity depends on selenium - breaks down H_2_O_2_ into water and lipid peroxides
**Non-enzymatic Antioxidants**	
Glutathione (GSH)	- serves as a scavenger of O_2_^•−^ and ^•^OH - essential cofactor for antioxidant enzymes - regenerates other oxidized antioxidants (vit C, vit E)
Uric acid	- protects against oxidative damage by scavenging O_2_, ^•^OH - strong electron donor and a selective scavenger of ONOO^−^
Phenolic compounds	- classified as primary antioxidants (capable of HAT (e.g., gallic acid, caffeic acid, epicatechin) and SET (e.g., kaempferol, resveratrol) - function as secondary oxidants due to the ability to bind to potentially pro-oxidative metal ions
Carotenoids	- react as antioxidant agents through three mechanisms: SET, the formation of one adduct, and HAT - excellent peroxyl radical scavengers
Vitamin C	- can produce reactions with oxidizing agents through HAT, SET or a concerted transfer of electron/protons (SET/HAT) - reacts with O_2_^•−^ and ^•^OH in the cytoplasm
Vitamin E	- prevents lipid peroxidation chain reactions and quenches O_2_ in cellular lipid compartments - reduces LOO^•^ by transferring the phenolic hydrogen atom of the chroman ring

O_2_^•−^, superoxide anion radical; H_2_O_2_, hydrogen peroxide; ^•^OH, hydroxyl radical; HAT, hydrogen-atom transfer; SET, single-electron transfer; LOO^•^, lipid peroxyl radicals; ONOO^−^, peroxynitrite anion; O_2_, molecular oxygen.

**Table 2:** Selected polyphenol compounds with antioxidant properties and their influence on the skin [21,23,35,36,40,41,42].

Compounds		Role as Antioxidants	Beneficial Effects on the Skin
Flavonoids	flavonols (e.g., quercetin, kaempferol, isorhamnetin)	interruption of free radical chain reactions; reducing properties (by donating an electron or a hydrogen atom); stabilization or delocalization of an unpaired electron leading to the formation of a stable phenoxyl radical; ability to chelate metal ions; ideal scavengers of superoxide radicals and inhibitors of lipid peroxidation	- can act as cofactors of enzymes - influence angiogenic and inflammatory processes - protect against radiation, moisturize and soften the skin - are used in anti-ageing, anti-cellulite, anti-couperose, and skin-lightening products
	flavones (e.g., apigenin, luteolin, rutin)		
	flavanols (e.g., catechin, epicatechin, procyanidins)		
	flavanones (e.g., naringenin, hesperetin)		
	anthocyanidins (e.g., cyanidin, peonidin, delphinidin)		
	isoflavonoids (e.g., genistin, daidzein)		
Phenolic acids	hydroxybenzoic acid derivatives (e.g., *p*-hydroxybenzoic, vanillic acid, protocatechuic acid)	predominantly radical scavenging via hydrogen atom donation; also electron donation and singlet oxygen quenching	- exhibit depigmenting properties by controlling the activity of tyrosinase - moisturize the skin and stimulate the synthesis of collagen and elastin fibres - anti-allergic, anti-cancer, anti-inflammatory, antimicrobial, anti-ageing properties - act as photoprotectors, prevent UV-induced erythema formation in the skin
	hydroxycinnamic acid derivatives (e.g., caffeic, *p*-coumaric, ferulic acids)		
Tannins	hydrolysable tannins	inhibition of lipid peroxidation and lipoxygenases in vitro, ability to scavenge radicals (e.g., hydroxyl, superoxide, and peroxyl)	- promote tropoelastin synthesis and reduce elastase activity - protect the skin from inflammation caused by external irritation - anti-inflammatory and wound healing properties - antimicrobial, cytotoxic, anti-cancer, antiulcer activity
	condensed tannins (proanthocyanidins)		
Stilbenes	trans-resveratrol and its glucoside	effective antioxidant in various in vitro assays, including total antioxidant activity, reducing power, DPPH^•^, ABTS^•+^, O_2_^•−^, H_2_O_2_ scavenging, and metal chelating; upregulate endogenous antioxidant pathways via activation of the Nrf2 pathway	- protect skin cells against oxidative damage caused by free radicals - anti-inflammatory effect - reduce hyperpigmentation (inhibit tyrosinase activity via suicide substrate type (Kcat) inhibition; affect post- transcriptional regulation of melanogenic genes; inhibit mRNA expression of TYR, TYR- related proteins 1 and 2, MITF and DCT in human melanocytes)

Nrf2, nuclear factor erythroid 2-related factor 2; TYR, tyrosinase; MITF, microphthalmia transcription factor; DCT, dopachrome tautomerase.

**Table 3:** Selected polyphenolic plant materials and their antioxidant properties **[33,34]**.

Plant	Botanical Name	Part Used	TPC (mg GAE/100 g DW)	TEAC (μmol TE/100 g DW)		
				ABTS	FRAP	DPPH
Thyme	*Thymus vulgaris*	herbal	0.58	35.4	693	295
Sage	*Salvia officinalis*	herbal	8.25	17.0	167	41.2
Lemon balm	*Melissa officinalis*	herbal	13.2	10.6	61.8	36.1
Clove	*Syzygium aromaticum*	fruit	8.96	346	2133	884
Sweet flag	*Acorus calamus*	rhizome	12.45	8.66	78.9	79.9
Yarrow	*Achillea millefolium*	herbal	9.55	11.2	191	200
Walnut	*Juglans regia*	leaf	0.24	27.3	128	119
Fig	*Ficus carica*	fruit	44.4	469	96.5	124
Grape red	*Vitis vinifera*	fruit	124	971	738	713
Tamarind	*Tamarindus indica*	fruit	318	2181	1171	1185
Prickly pear	*Opuntia ficus-indica*	fruit	43.7	472	207	122
Hops	*Humulus lupulus*	cone	7.14	10.8	50.3	83.2
Purple coneflower	*Echinacea purpurea*	leaf	15.15	12.3	94.6	75.0
Nutmeg	*Myristica fragrans*	fruit	8.95	33.3	218	182
Cinnamon	*Cynamonum zeylanicum*	seed	0.13	140	233	253
Passion fruit	*Passiflora edulis*	fruit	292	3680	1883	1125
Turmeric	*Curcuma longa*	rhizome	1.72	19.5	62.6	100
Apricot	*Prunus armeniaca*	fruit	20.6	144	77.7	72.7
Licorice	*Glycyrrhiza glabra*	herbal	1.15	30.8	67.3	177
Jujube	*Ziziphus jujuba*	fruit	400	1618	1724	980
Apple	*Malus domestica*	fruit	197	142	92.5	52.9
Peach	*Prunus persica*	fruit	53.2	104	37.1	46.5
Cherry	*Prunus avium*	fruit	70.2	640	389	242
Papaya	*Carica papaya*	fruit	56	316	458	234
Pineapple	*Ananas comosus*	fruit	56.2	590	379	202
Pomegrante	*Punica granatum*	fruit	133	1478	1180	987
Parsley	*Petroselinum sativum*	root	2.02	11.8	40.9	39.9
Milk thistle	*Silybum marianum*	seed	4.77	12.3	65.7	34.3
Raspberry	*Rubus idaeus*	fruit	266	4628	3927	2150
Strawberry	*Fragaria vesca*	fruit	131	1629	1153	1053
Blackberry	*Rubus fruticosus*	fruit	301	4998	3995	2210
Blueberry	*Vaccinium corymbosum*	fruit	258	4023	2390	1456
Redcurrant	*Ribes sativum*	fruit	269	4063	3177	1927

TPC, total phenolic content expressed as gallic acid equivalent (GAE); TEAC, trolox equivalent antioxidant capacity; TE, trolox equivalent; DW, dry weight; ABTS, 2,2’-azinobis-(3-ethylbenzothiazoline-6-sulfonic acid); FRAP, ferric reducing antioxidant power; DPPH, 2,2’-diphenyl-1-picrylhydrazyl.

**Table 4:** Antioxidant activity of dominant carotenoids present in human skin [58,59,60,61,62].

Compound	Main Effects
β-Carotene	- directly scavenges ROS - ↑ GPx, CAT, GST and vit C - protects liquid crystal lipid structures from UVR - inhibits UVR-induced proline oxidation in collagen - protects lipids in the intercellular matrix from oxidation - inhibits oxidant-induced NF-kB activation and IL-6 and TNF-α production - protects the immune system from damage by UVA - suppresses UVA induction of MMP-1, MMP-3, and MMP-10 involved in photoageing - blocks ^1^O_2_-mediated induction of MMP-1 and MMP-10
Lycopene	- ↓ production of ROS and protects cells against OxS - antioxidant activity based on hydrogen transfer reactions - quenches radicals in the hydrophobic part of the membrane - can eliminate ROO^•^, thereby inhibiting LPO - more effective than β-carotene in protecting cells against H_2_O_2_ - stabilizes other antioxidants, such as vit C and vit E
Lutein	- protects cell membranes against oxidative damage - effective as an antioxidant in the polar region - may reduce LPO and quench ^1^O_2_ - prevents the decrease in CAT and SOD - protects the fibroblasts from UVA-induced oxidative action - protects against UVB-induced skin damage, photoageing and photocarcinogenesis

↑, enhances; ↓, reduces; ROS, reactive oxygen species; GPx, glutathione peroxidase; CAT, catalase; GST, glutathione transferase; UVR, ultraviolet radiation; NF-κB, nuclear factor kappa-light-chain-enhancer of activated B cells; IL-6, interleukin 6; TNF-α, tumour necrosis factor α; MMP, metalloproteinase; OxS, oxidative stress; ^1^O_2,_ singlet oxygen; ROO^•^, peroxyl radicals; LPO, lipid peroxidation; H_2_O_2,_ hydrogen peroxide; SOD, superoxide dismutase.

**Table 5:** Major natural compounds, and antioxidant activity of selected EOs.

Species (Family)	Part Used	Main Constituents of EOs	AA IC_50_ (μg/mL)	References
*Cinnamomum zeylanicum* *(Lauraceae)*	bark	cinnamyl aldehyde (45.13%), cinnamyl alcohol (5.13%), eugenol (7.47%), methyl-eugenol (5.23%), ethyl-cinnamate (3.86%), dihydro-eugenol (3.31%)	13.10	[116]
*Carum carvi* *(Apiaceae)*	fruits	carvone (48.53%), limonene (44.42%)	46.51	[117]
*Citrus limon* *(Rutaceae)*	flowers	β-pinene (25.44%), limonene (39.74%), linalool (2.16%), α-terpineol (7.30%), linalyl acetate (3.01%), acetate geranyl (3.03%), nerolidol (6.91%), acetate neryl (1.74%), farnesol (4.28%).	15.06	[118]
*Eugenia caryophyllata* *(Myrtaceae)*	flower buds	eugenol (77.61%), eugenol acetate (6.54%), psi-cumene (3.03%), prehnitene (2.73), β-*cis*-caryophyllene (2.53%)	30.27	[119]
*Melaleuca alternifolia* *(Myrtacea)*	aerial parts	terpinene-4-ol (31.11%), γ-terpinene (25.30%), α-terpinene (12.70%), 1,8-cineole (6.83%), *p*-cymene (4.23%), terpinolene (4.03%), limonene (2.50%), α-terpineol (2.35%), aromadendrene (1.75%), δ-cadinene (1.41%)	48.35	[120]
*Mentha spicata* *(Lamiaceae)*	aerial parts	menthol (69.05%), l-menthone (12.21%), l-menthyl acetate (3.73%), (+)-isomenthone (3.07%), neoisomenthol (1.63%), d-limonene (1.53%),	23.95	[121]
*Ocimum basilicum* *(Lamiaceae)*	leaves	trans-β-guaiene (16.89%), α-cadinol (15.66%), 9-methoxybicyclo [6.1.0] nona-2, 4, 6-triene (11.36%), phytol (11.68%), eucalyptol (3.03%)	13.21	[122]
*Origanum vulgare* *(Lamiaceae)*	aerial parts	carvacrol (20.82%), thymol (14.64%), *p*-cymene (14.11%),1-methoxy-4-methylbenzene (11.34%), γ-terpinene (6.14%), 2-isopropyl-5-methylanisole (5.6%), 3-octanol (2.05%), 1-octen-3-ol (1.66%)	1.47	[121]
*Perilla frutescens* *(Lamiaceae)*	aerial parts	perillaketone (35.56%), isoegomaketone (20.40%), caryophyllene (10.21%), (Z,E)-α-farnesene (4.44%), isoelemicin (3.29%)	3.77	[121]
*Piper nigrum* *(Piperaceae*)	seeds	β-caryophyllene (18.64%), limonene (14.95%), sabinene (13.19%), 3-carene (8.56%), β-pinene (9.71%), and α-pinene (7.96%)	16.27	[123]
*Pogostemon cablin* *(Lamiaceae)*	aerial parts	patchouli alcohol (50.52%), α-guaiene (6.09%), α-bulnesene (5.68%), pogostone (5.45%), pogostol (5.43%), caryophyllene oxide (1.86%), β-patchoulene (1.23%), ledol (1.22%)	49.74	[121]
*Rosmarinus officinalis* *(Lamiaceae)*	leaves	eucalyptol (11.31%), verbenone (16.56%), α-pinene (26.46%), geraniol (5.91%)	20.36	[121]
*Salvia officinalis* *(Lamiaceae)*	aerial parts	tanacetone (27.99%), camphor (16.21%), viridiflorol (7.85%), humulene (6.44%), eucalyptol (5.11%)	11.86	[121]
*Thymus mongolicus* *(Lamiaceae)*	aerial parts	thymol (23.7%), *p*-cymene (21.17%), γ-terpinene (16.42%), β-bisabolene (3.96%), linalool (2.97%), carvacrol (2.48%), α-terpinene (2.44%), thymol acetate (2.22%)	1.42	[121]
*Zingiber officinale* *(Zingiberaceae)*	rhizomes	β-sesquiphellandrene (27.16%), caryophyllene (15.29%), zingiberene (13.97%), α-farnesene (10.52%), ar-curcumene (6.62%)	65.5	[116]

AA, antiradical activity is expressed as IC_50_ (μg/mL), the concentration required to cause 50% DPPH inhibition; the lower the IC_50_ value, the higher the antioxidant activity of EOs.

**Table 6:** Selected antioxidants of plant origin, some of their common chemical constituents, and their importance for skin.

**Plant Name**	**Part Used**	**Key Chemical Constituents**	**Activity**	**References**
Aloe vera *Aloe barbadensis*	leaf	vitamins (A, C, E), minerals, amino acids, enzymes, polysaccharides, saponins, anthraquinones, lignin, salicylic acid palmitic acid, oleic acid, caprylic acid, stearic acid, β-sitosterol	antioxidant activity, prevents UVR-induced skin damage; moisturizing, antipruritic, astringent, soothing and cooling effect; antimicrobial, antifungal, wound healing, and anti-inflammatory activity	[125,137]
Amla *Emblica officinalis*	fruit	ascorbic acid, minerals, calcium, iron, amino acids, carotenes; polyphenols, e.g., phyllembin, flavonoids, kaempferol	antioxidant activity, free radical scavenging, UV protection; promotes procollagen production	[138,139,140]
Brazil nut *Bertholletia excelsa*	seed	fatty acids (75% unsaturated fatty acids, mainly oleic and linoleic acids), phytosterols, phenolic compounds, vit E, selenium	antioxidant properties; protects against free radicals; treatment of dry, flaky, and ageing skin, acne, skin inflammation	[141,142]
Chamomile *Matricaria recutita*	flower	flavonoids (apigenin, luteolin, patuletin-7-glycosides), coumarins (umbelliferone and herniarin)	antioxidant, anti-inflammatory, antibacterial activity; soothes irritated skin; treatment of atopic dermatitis	[3,143,144,145]
Chokeberry *Aronia melanocarpa*	fruit	anthocyanins (cyanidin-3-arabinoside, cyanidin-3-galactoside, cyanidin-3-glucoside, cyanidin-3-xyloside, pelargonidin-3-arabinoside), flavonols (quercetin derivatives, kaempferol), flavan-3-ols (epicatechin), hydroxycinnamic acids (chlorogenic acid, neochlorogenic acid)	antioxidant and anti-inflammatory properties, beneficial effects on skin, especially in prevention of premature skin ageing and wrinkling	[146,147]
Elderberry *Sambucus nigra*	flower	flavonoids: quercetin, isoquercetin, kaempferol, myricetin, rutin, nicotiflorin and their glycosides; phenolic acids: caffeic, chlorogenic, p-coumaric, ferulic	antioxidant, astringent, anti-inflammatory, antibacterial, capillary stabilizing properties	[148,149,150]
European cranberry *Vaccinium oxycoccos*	fruit	anthocyanins (cyanidin glycosides, peonidins, delphinidins, malvidins, petunidins); flavonols (quercetin, myricetin, kaempferol), resveratrol	antioxidant, anti-inflammatory, anti-allergic, capillary stabilizing, anti-ageing activity	[151]
French maritime pine *Pinus pinaster*	bark	phenolic compounds e.g., polyphenolic monomers, procyanidins, and phenolic acids (derivatives of benzoic and cinnamic acids)	potent scavenger of free radicals, protects against OxS, improves skin conditions, including chronic venous insufficiency and skin inflammation, hydration, and elasticity (increased synthesis of ECM); wound healing activity	[152,153]
Ginger *Zingiber officinale*	root	gingerols and shogaol, organic acids (oxalic and tartaric acids); essential oils (major components: camphene, sabinene, *p*-cineole, α-terpineol, α-curcumene, zingiberene, α-farnesene, β-sesquiphellandrene, neral, geranial)	antioxidant effect nearly equal to that of synthetic antioxidants, including BHA and BHT, prevents free radical generation, reduces OxS; antibacterial and anti-fungal activity	[154,155,156]
Ginkgo *Ginkgo biloba*	leaf	flavonoids, terpenoids (ginkgolides, bilobalide), proanthocyanids, organic acids, tannins, sitosterols, carotenoids, polysaccharides	antioxidant and anti-inflammatory properties, smooths and rejuvenates skin, improves skin microcirculation, elasticity and hydration, promotes fibroblast growth, increases the production of collagen and fibronectin; protects against UVR damage	[157,158,159,160]
Grapes *Vitis vinifera*	fruit	oligomeric proanthocyanidins; phenolic acids: cinnamic acids (coumaric, caffeic, ferulic, chlorogenic, and neochlorogenic acid) and benzoic acids (*p*-hydroxybenzoic, protocatechuic, vanillic, and gallic acid); flavonoids: flavan-3-ols (catechin, epicatechin, and their polymers), flavanones (quercetin)	antioxidant activity (stronger than vit C and vit E), facilitation of wound healing, protection of collagen and elastin from degradation, tyrosinase-inhibiting activity	[30,129,161]
Green tea *Camellia sinensis*	leaf	flavandiols, catechins (especially epigallocatechin-3-gallate (EGCG), flavonols, phenolic acids	antioxidant (20 times stronger than vit E); ability to heal UV photo-damage and phototoxicity; stimulates the formation of ceramides and sphingolipids in the skin; treatment of atopic dermatitis; anti-inflammatory, antimicrobial, and anti-ageing activity	[2,3,30,127,162]
Hawthorn *Crataegus monogyna*	fruit, flower	chlorogenic acid, epicatechin, hyperoside, isoquercitrin, protocatechuic acid, quercetin, rutin, ursolic acid	antioxidant and antimicrobial activity, toning action on skin tissue, anti-wrinkle, skin hydration	[163]
Hibiscus *Hibiscus sabdariffa*	flower	phenolic compounds (including anthocyanins, protocatechuic acid), vit E	antioxidant, antibacterial and anti-inflammatory activity, skin care, skin protection, anti-ageing	[141]
Lavender *Lavandula officinalis*	flower leaf	essential oil monoterpenoids (including linaloyl-acetate, linalool, 1-terpinen-4-ol), leaves contain rosmarinic acid, tannins, coumarins, triterpenes and phenolic acids	wound healing, antioxidant, antibacterial, and antimicrobial activity	[143,145]
Licorice *Glycyrrhiza glabra*	root	glycoside glycyrrhizin, glycyrrhetinic acid, flavonoids, isoflavonoids, chalcones	skin whitening, antioxidant, antimicrobial, and anti-inflammatory properties; treatment of skin irritations, dermatitis, eczema, acne, sunburn	[30,163,164]
Marigold *Calendula officinalis*	flower	polyphenols, including rutin and narcissin, esculetin, quercetin-3-*O*-glucoside	antioxidant, anti-inflammatory, antibacterial, antiviral, antifungal activities; prevents UV irradiation-induced OxS in skin, treatment of wounds, burns, dermatitis	[130,162]
Milk thistle *Silybum marianum*	fruit	flavonoids-silymarin (silybin, silidianin, and silicristin)	antioxidant, reduction of UV-induced immune suppression, OxS, sunburn cell formation and apoptosis, anti-tumour effect	[67,125]
Olive *Olea europaea*	leaf, fruit	phenolic compounds: hydroxytyrosol, tyrosol in fruits, oleuropein, luteolin 7-*O*-glucoside in leaves	antioxidant, antimicrobial, anticarcinogenic, anti-inflammatory activities, improves skin texture and integrity, moisturizes skin	[144,163,165]
Pepper *Piper longum*	fruit	volatile oils, alkaloid piperine and piperettine	antioxidant potency in vitro and in vivo; used topically in a cream base to treat sunburn	[138]
Pomegranate *Punica granatum*	fruit	vit C and K, polyphenols such as ellagitannins, punicalagins, granatin A and B, punicacotein A, B, C, punicafolin, punigluconin, punicalagin, punicalin	protection of human immortalized HaCaT keratinocytes against UVB-induced OxS and markers of photoageing	[131,137]
Purple coneflower *Echinacea purpurea*	root	polyphenols, alkylamides, polysaccharide	antioxidant activity, protects collagen against free radical damage; anti-inflammatory, antiviral, antimicrobial, antiproliferative effects	[166,167,168]
Red clover *Trifolium pretense*	flower	isoflavones (equol)	antioxidant, sun-protective cosmetic ingredient; treatment of psoriasis, eczema, acne	[132]
Rosemary *Rosmarinus officinalis*	leaf	flavonoids (including luteolin, genkwanin, hesperidin, eriocitrin, isorhamnetin, diosmin and their glycosides), phenolic acids (rosmarinic acid, caffeic acid, chlorogenic acid, ferulic acid), carnosic acid, carnosol	antioxidant, anti-inflammatory, antibacterial, anti-wrinkle, and firming effects; stimulates circulation	[169,170,171]
Safflower *Carthamus tinctorius*	seed	flavonoid hydroxysafflor yellow A	antioxidant, anti-ageing, anti-inflammatory activity; inhibits melanogenesis and apoptosis; improves diabetic wound healing	[172,173]
Sea buckthorn *Hippophaë rhamnoides*	fruit	flavanols and flavonols (isoramnetin, quercetin, myricetin and kaempferol), proanthocyanidins and phenolic acids (*m*-, *o*- and *p*-coumaric, caffeic, ferulic, sinapinic, gallic, ellagic and cinnamic acids)	antioxidant, moisturizing, and revitalizing effects; regulation of sebum secretion (inhibits the action of 5-α reductase type 1, an enzyme active in the sebaceous glands); promotes wound healing and the synthesis and stabilization of collagen	[85,174,175]
Soybean *Glycine max*	seed	isoflavones (genistein, daidzein)	significantly inhibits oxidative damage; photoprotective, DNA-protective and antiphotocarcinogenic properties; antipigmentary capabilities; boosts hyaluronic acid levels in skin	[133,134,176]
Sponge gourd *Luffa cylindrica*	seed leaf	unsaturated fatty acids (stearic and linoleic acids), phenolic compounds glycosides, tannins, flavonoids, saponins	free radical scavenging properties; inhibits generation of free radicals significant scavenging of DPPH and H_2_O_2_ radical	[138,177,178] [179]
St. John’s Wort *Hypericum perforatum*	herb	tannins and flavonoids; hyperforin; naphthodianthrone hypericin	anti-inflammatory, anti-tumour and antibacterial properties; anti-oxidative properties, reduces free radical formation in the skin after exposure to UV and IR radiation; treatment of wounds, burns, eczema	[135,180]
Tea tree *Melaleuca alternifolia*	leaf	EOs, 1, 8-cineole, terpinen-4-ol	antioxidant and anti-inflammatory activity, broad-spectrum antibacterial, antiviral, and antifungal activity, relieves sunburn, treatment of acne, seborrheic dermatitis, warts, burns	[30,67,181]
Turmeric *Curcuma longa*	rhizome	curcuminoids: curcumin (71.5%), demethoxycurcumin (19.4%), and bisdemethoxycurcumin (9.1%); zingiberene	antibacterial, antioxidant, anti-inflammatory properties, used for prevention, treatment or control of psoriasis and other skin conditions such as acne, rosacea, wounds, burns, eczema, photodamage, premature ageing	[30,144,182,183]
Walnut *Juglans regia*	leaf	juglone (5-hydroxy-1,4-naphthoquinone)	antioxidant activity, prevents oxidative damage; UV protection properties; self-tanning sunscreen agent	[125,136]

## Data Availability

Not applicable.

## Abbreviations

^•^OH	hydroxyl radical
^1^O_2_	singlet oxygen
4-HNE	4-hydroxynonenal
8-OHdG	8-hydroxy-20-deoxyguanosine
AA	antiradical activity
ABTS	2,2’-azinobis-(3-ethylbenzothiazoline-6-sulfonic acid)
BHA	butylated hydroxyanisole
BHT	butylated hydroxytoluene
BPs	bioactive peptides
CAA	cellular antioxidant activity
CAT	catalase
CUPRAC	cupric ion reducing antioxidant capacity
DCT	dopachrome tautomerase
DMBA	7,12-dimethylbenz[a]anthracene
DPPH	2,2’-diphenyl-1-picrylhydrazyl
ECM	extracellular matrix
EOs	essential oils
ERKs	extracellular signal-regulated kinases
FRAP	ferric antioxidant power reduction
GSH	glutathione
GPx	glutathione peroxidase
GR	glutathione reductase
GST	glutathione transferase
H_2_O_2_	hydrogen peroxide
HAT	hydrogen-atom transfer
IL-6	interleukin 6
IP-10	interferon γ-induced protein 10
I-TAC	interferon-inducible T-cell α chemoattractant
JNKs	c-Jun N-terminal kinases
LOO^•^	lipid peroxyl radicals
LPO	lipid peroxidation
MAPK	mitogen-activated protein kinase
M-CSF	macrophage colony-stimulating factor
MDA	malondialdehyde
MED	minimal erythema dose
MIG	monokine induced by interferon-gamma
MITF	microphthalmia transcription factor
MMP	matrix metalloproteinase
NF-κB	nuclear factor kappa-light-chain-enhancer of activated B cells
Nrf2	nuclear factor erythroid 2-related factor 2
NO	nitric oxide
O_2_^•−^	superoxide anion radical
ONOO⁻	peroxynitrite anion
OxS	oxidative stress
PM	phosphomolybdenum
RNS	reactive nitrogen species
RO^•^	alkoxyl radicals
ROO^•^	peroxyl radicals
ROOH	organic hydroperoxides
ROS	reactive oxygen species
SET	single-electron transfer
SOD	superoxide dismutase
TAS	total antioxidant status
TBARS	thiobarbituric acid reactive substances
TIMP-2	tissue inhibitor of metalloproteinase 2
TNF-α	tumour necrosis factor α
TPA	12-O-tetradecanoyl phorbol-13-acetate
TPC	total phenolic content
TRP1	tyrosine-related protein 1
TRX	thioredoxin
TYR	tyrosinase
UVR	ultraviolet radiation
VCAM-1	vascular cell adhesion molecule 1
